# The burden of cardiovascular diseases attributable to metabolic risk factors and its change from 1990 to 2019: a systematic analysis and prediction

**DOI:** 10.3389/fepid.2023.1048515

**Published:** 2023-05-25

**Authors:** Huaigen Wang, Jing Liu, Yunfei Feng, Aiqun Ma, Tingzhong Wang

**Affiliations:** ^1^ Department of Cardiovascular Medicine, The First Affiliated Hospital of Xi’an Jiaotong University, Xi’an, China; ^2^ Shaanxi Key Laboratory of Molecular Cardiology, Xi'an, China; ^3^ Key Laboratory of Environment and Genes Related to Diseases, Xi’an Jiaotong University, Ministry of Education, Xi’an, China

**Keywords:** cardiovascular diseases, metabolic risk factors, global disease burden, forecast, hypertension

## Abstract

**Background:**

Metabolic disorders are the most important risk factors for cardiovascular diseases (CVDs). The purpose of this study was to systematically analyze and summarize the most recent data by age, sex, region, and time, and to forecast the future burden of diseases.

**Methods:**

Data on the burden of CVDs associated with metabolic risk factors were obtained from the Global Burden of Disease (GBD) Study 2019; and then the burden of disease was assessed using the numbers and age-standardized rates (ASR) of deaths, years of life lost (YLLs), years of life lived with disability (YLDs), and disability-adjusted life-years (DALYs) and analyzed for temporal changes, differences in age, region, sex, and socioeconomic aspects; finally, the burden of disease was predicted using an autoregressive integrated moving average (ARIMA) model.

**Results:**

From 1990 to 2019, the numbers of deaths, DALYs, YLDs, and YLLs attributed to metabolic risk factors increased by 59.3%, 51.0%, 104.6%, and 47.8%, respectively. The ASR decreased significantly. The burden of metabolic risk factor-associated CVDs was closely related to socioeconomic position and there were major geographical variations; additionally, men had a significantly greater disease burden than women, and the peak shifted later based on the age group. We predicted that the numbers of deaths and DALYs would reach 16.5 million and 324.8 million, respectively, by 2029.

**Conclusions:**

The global burden of CVDs associated with metabolic risk factors is considerable and still rising, and more effort is needed to intervene in metabolic disorders.

## 1. Introduction

Approximately 523 million people worldwide suffered from cardiovascular disease (CVDs) in 2019, and CVDs have also become the leading cause of disability and death (1–3). Risk factors for CVDs include metabolic, environmental, and behavioral aspects. Some risk factors for CVDs, such as age, gender, and genetics, cannot be modified, but many others, including hypertension, obesity, diabetes, dyslipidemia, smoking, and air pollution, can be prevented or corrected (4–6). Over the past several decades, the treatment and intervention of hypertension, diabetes, and other disorders have significantly decreased the incidence of cardiovascular disease, death, and disability (7, 8). Nonetheless, it is worth noting that as the world's population has aged and the number of obese people has increased, the risk of death and disability from cardiovascular disease has remained high in most regions, and the number of deaths caused by cardiovascular disease has been constantly increasing (9).

According to the data from the Global Burden of Disease Study 2019 (GBD 2019), while some environmental and behavioral risk factors showed a decline, metabolic risk factors increased significantly from 2010 to 2019 (10), and the influence of metabolic factors (including hypertension, diabetes, obesity, dyslipidemia, and kidney dysfunction) on the occurrence, development, and prognosis of cardiovascular disease is becoming increasingly important (11–14). It is estimated that in 2019, 42% of female deaths and 51% of male deaths were related to metabolic risk factors (10). Nearly all metabolic risk factors are preventable or modifiable; therefore, it is essential to evaluate and summarize their role in the global burden of CVDs and their changing trends. However, recent studies on the impact of metabolic risk factors on the burden of CVDs are lacking. In this study, we provide a comprehensive analysis in terms of time, age, sex, region, and socioeconomic status, and we forecast future illness burden to provide fresh insights into the prevention and treatment of CVDs.

## 2. Methods

### 2.1. Data sources

GBD 2019 was an integrated surveillance system that used comparative risk assessment (CRA) to estimate attributable mortality, years of life lost (YLLs), years of life lived with disability (YLDs), and disability-adjusted life-years (DALYs) at the global and regional levels for 204 countries and territories from 1990 to 2019. The Global Health Data Exchange (GHDx) query tool (https://ghdx.healthdata.org/gbd-results-tool) was used to obtain data on yearly metabolsim-related numbers and age-standardized rates (ASR) of CVDs deaths, DALYs, YLLs, YLDs by location, age, sex, and socioeconomic status from 1990 to 2019.

### 2.2. Definition

In this study, CVDs consisted of ischemic heart disease, aortic aneurysm, rheumatic heart disease, stroke, hypertensive heart disease, nonrheumatic valvular heart disease, cardiomyopathy and myocarditis, atrial fibrillation and flutter, aortic aneurysm, endocarditis, peripheral arterial disease and other cardiovascular and circulatory diseases. The metabolic risk factors included high fasting plasma glucose (FBG), high low-density lipoprotein cholesterol (LDL-C), high systolic blood pressure (SBP), high body-mass index (BMI), and kidney dysfunction.

### 2.3. Statistical analysis

The global burden of CVDs attributable to metabolic risk factors is represented by the number and ASR and their 95% uncertainty intervals (95% UI) of deaths, DALYs, YLLs, and YLDs. The age-standardized rate (ASR) is a measure of the rate a population would have if it had a standard age structure. Standardization is required when comparing populations of different ages because age has a substantial impact on the risk of dying from CVDs. The ASR was calculated as follows: $ASR=\frac{\sum_{i=1}^{A}a_{i}w_{i}}{\sum_{i=1}^{A}w_{i}}\times 100,000$, where $a_{i}$ is specific age ratio, $w_{i}$ is the number (or weight) of the selected standard population,and 100,000 indicates per 100,000 population (15).

EAPC is a concise and frequently utilized metric of the ASR trend over a given time period. With the following equation: y=α+βx+ε, the natural logarithm of the regression line is fitted to the ASR, where y=ln⁡(ASR), and x=calendaryear. Using the linear regression model, the estimated annual percentage change (EAPC) and its 95% confidence interval (CI) were calculated as 100×[exp⁡(β)−1] (16). An EAPC of 0, a positive EAPC and a negative EAPC show that the rate is constant, has a downward trend, or has an upward trend over time, respectively; the greater the absolute value of the EAPC is, the more rapidly the rate changes over time. The sociodemographic index (SDI), which ranges from 0 (worst) to 1 (best), is a lagged distribution of the total fertility rate of the population under 25, the average education level of adults, and per capita income indices. The EAPC and its 95% confidence interval (CI) were used to quantify temporal trends (17).

In order to understand the future trends of the CVDs burden attributed to metabolic risk factors, we used the Autoregressive integrated moving average (ARIMA) model to make predictions in this study. ARIMA is a commonly used time series model, where p, d, and q are the order of autoregression (AR), difference and partial autoregression (MA) required to make the data stationary, respectively. The ARIMA model consists of the following three main components: AR refers to a model that shows a changing variable that regresses on its own lagged, or prior, values, integrated (I) represents the differencing of raw observations to allow for the time series to become stationary, MA incorporates the dependency between an observation and a residual error from a moving average model applied to lagged observations (18). The ARIMA model is built using “auto.arima” functions from the “forecast” and “tseries” packages. The optimal model and parameters are selected in accordance with the Akaike Information Criterion (AIC) and Bayesian Criterion (BIC). The Ljung-Box test is conducted on the model's residual sequence. If *P *> 0.05, the test is passed, indicating that the data are not white noise and that the ARIMA model is well-fitting; otherwise, the model is remodeled (19). The constructed model was then used to predict the number and ASRs of deaths, DALYs, YLDs, and YLLs per year until 2029. All statistical analyses were carried out using R (version 4.1.3).

## 3. Results

### 3.1. Metabolic risk factors related to CVDs

**Figure 1:**
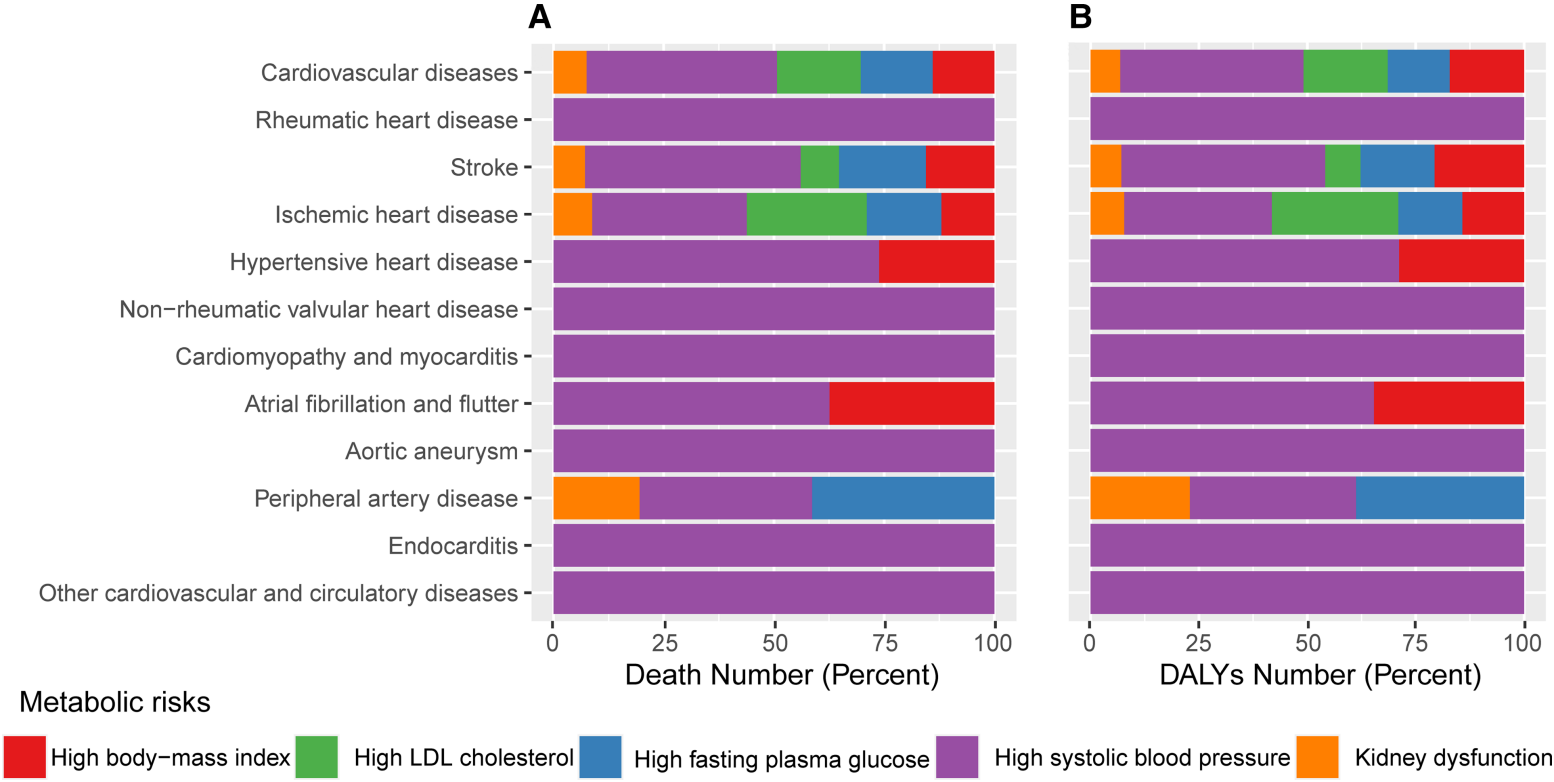
The proportion of each CVDs attributed to individual metabolic risk factors in 2019. (**A**) Proportions of CVDs deaths due to individual metabolic risk factors. (**B**) Proportions of CVDs DALYs due to individual metabolic risk factors. CVDs, cardiovascular diseases; DALY, disability-adjusted life year.

High SBP, followed by high LDL-C, high FBG, high BMI, and renal dysfunction, were the metabolic risk factors with the biggest effects on CVDs (Figure 1). Elevated SBP was linked to all types of CVDs and causes 43.2 percent of deaths number (10.0 million) and 42.2 percent of DALYs number (213.9 million) attributable to metabolic abnormalities in the CVDs in 2019. The second risk factor with the largest effects was high LDL-C and was responsible for 19.0 percent of metabolic-related CVDs mortality and 19.5 percent of DALYs number. Increased BMI was closely related to cardiovascular disorders such stroke, ischemic heart diseases, hypertensive heart diseases, and AF and caused 14.0% of deaths and 17.1% of DALYs number (Supplementary Table S1).

### 3.2. Change in the global burden of CVDs attributable to metabolic risk factors from 1990 to 2019

**Figure 2:**
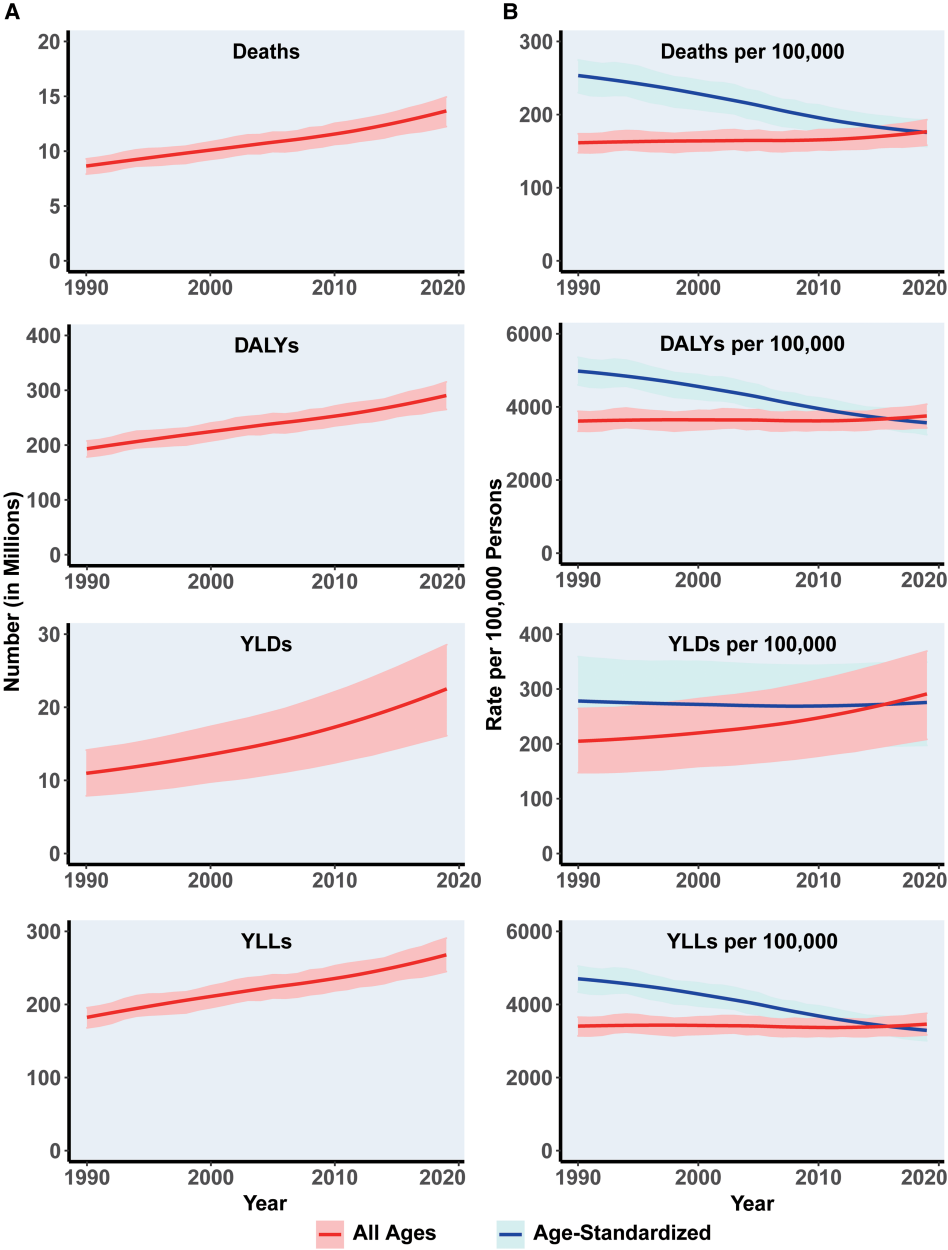
The global burden change in metabolism-related CVDs globally between 1990 and 2019. (**A**) Total number of cardiovascular disease Deaths, DALYs, YLDs, and YLLs due to metabolic risk. Shaded regions represent 95% uncertainty intervals. (**B**) Age-standardized and all-ages Deaths, DALY, YLD, and YLLs rates of metabolism-related CVDs. Shaded regions represent 95% uncertainty intervals. CVDs, cardiovascular diseases; ASR, age standardized rate; DALYs, disability-adjusted life years; YLDs, years lived with disability; YLLs, years of life lost.

From 1990 to 2019, the global burden of CVDs attributed to metabolic risk factors continued to rise (Figure 2). Specifically, the numbers of deaths, DALYs, YLDs, and YLLs increased by 59.3%, 51.0%, 104.55%, and 47.8%, respectively. CVDs deaths and DALYs number attributable to metabolic risk factors reached 13.7 million and 192.7 million, respectively, in 2019. The ASR of CVDs attributable to metabolic risk factors showed a significant downward trend after taking into account the effects of increasing population as well as aging. The ASRs for deaths, DALYs, YLDs, and YLLs decreased from 252.9 [95% UI (230.0, 274.6)] to 176.1 [95% UI (156.2, 192.4)], 4,965.8 [95% UI (4,602.7, 5,357.3)] to 3,573.4 [95% UI (3,240.3, 3,870.5)], 278.9 [95% UI (199.9, 359.0)] to 274.9 [95% UI (197.2, 349.1)], and 4,686.9 [95% UI (4,341.0, 5,048.4)] to 3,298.6 [95% UI (3,007.1, 3,572.7)] per 100,000 people, respectively (Table 1). The EAPCs of deaths and DALYs ASR were −1.4 (−1.4,1.3) and −1.2 (−1.3,−1.2), respectively (Supplementary Table S2).

**Table 1:** Number and ASR of global metabolism-related CVDs outcomes between 1990 and 2019.

Outcomes		Gender	Number	95% UI		ASR	95% UI	
				Lower	Upper		Lower	Upper
1990	Deaths	Both	8,610,099	7,912,192	9,294,283	252.9	230.0	274.6
		Male	4,269,219	3,929,525	4,617,849	283.3	259.4	307.3
		Female	4,340,880	3,904,069	4,737,601	225.7	201.2	247.0
	DALYs	Both	192,671,144	178,356,725	207,535,041	4,965.8	4,602.7	5,357.3
		Male	105,386,374	96,947,180	113,952,326	5,795.0	5,345.1	6,264.5
		Female	87,284,770	79,671,422	94,849,549	4,190.2	3,823.6	4,557.1
	YLDs	Both	10,989,629	7,889,220	14,147,669	278.9	199.9	359.0
		Male	4,821,239	3,438,111	6,208,653	265.0	190.6	341.4
		Female	6,168,391	4,439,424	7,879,304	289.7	208.3	370.8
	YLLs	Both	181,681,514	168,010,920	195,393,075	4,686.9	4,341.0	5,048.4
		Male	100,565,135	92,489,541	108,778,276	5,530.0	5,095.0	5,989.0
		Female	81,116,379	74,260,453	88,076,063	3,900.5	3,563.4	4,241.4
2019	deaths	Both	13,707,277	12,241,260	14,937,445	176.1	156.2	192.4
		Male	7,134,695	6,408,188	7,800,898	205.3	183.1	224.8
		Female	6,572,582	5,728,970	7,367,302	149.9	130.7	168.0
	DALYs	Both	290,946,991	265,032,973	315,180,527	3,573.4	3,240.3	3,870.5
		Male	166,092,792	150,740,371	180,861,047	4,327.9	3,922.9	4,713.6
		Female	124,854,199	111,781,261	138,136,289	2,865.5	2,565.9	3,170.5
	YLDs	Both	22,469,257	16,115,957	28,563,138	274.9	197.2	349.1
		Male	10,212,010	7,336,634	13,182,379	265.5	190.3	343.2
		Female	12,257,247	8,720,055	15,561,499	282.4	200.7	358.2
	YLLs	Both	268,477,734	245,012,615	290,699,760	3,298.6	3,007.1	3,572.7
		Male	155,880,781	141,138,649	169,865,667	4,062.3	3,681.3	4,428.8
		Female	112,596,953	100,429,824	125,153,209	2,583.1	2,303.9	2,870.6

ASR (per 100,000 population), age standardized rate; CVDs, cardiovascular disease; UI, uncertainty interval; DALYs, Disability-Adjusted Life Years; YLDs, Years Lived with Disability; YLLs, Years of Life Lost.

### 3.3. Regional differences in variation

**Figure 3:**
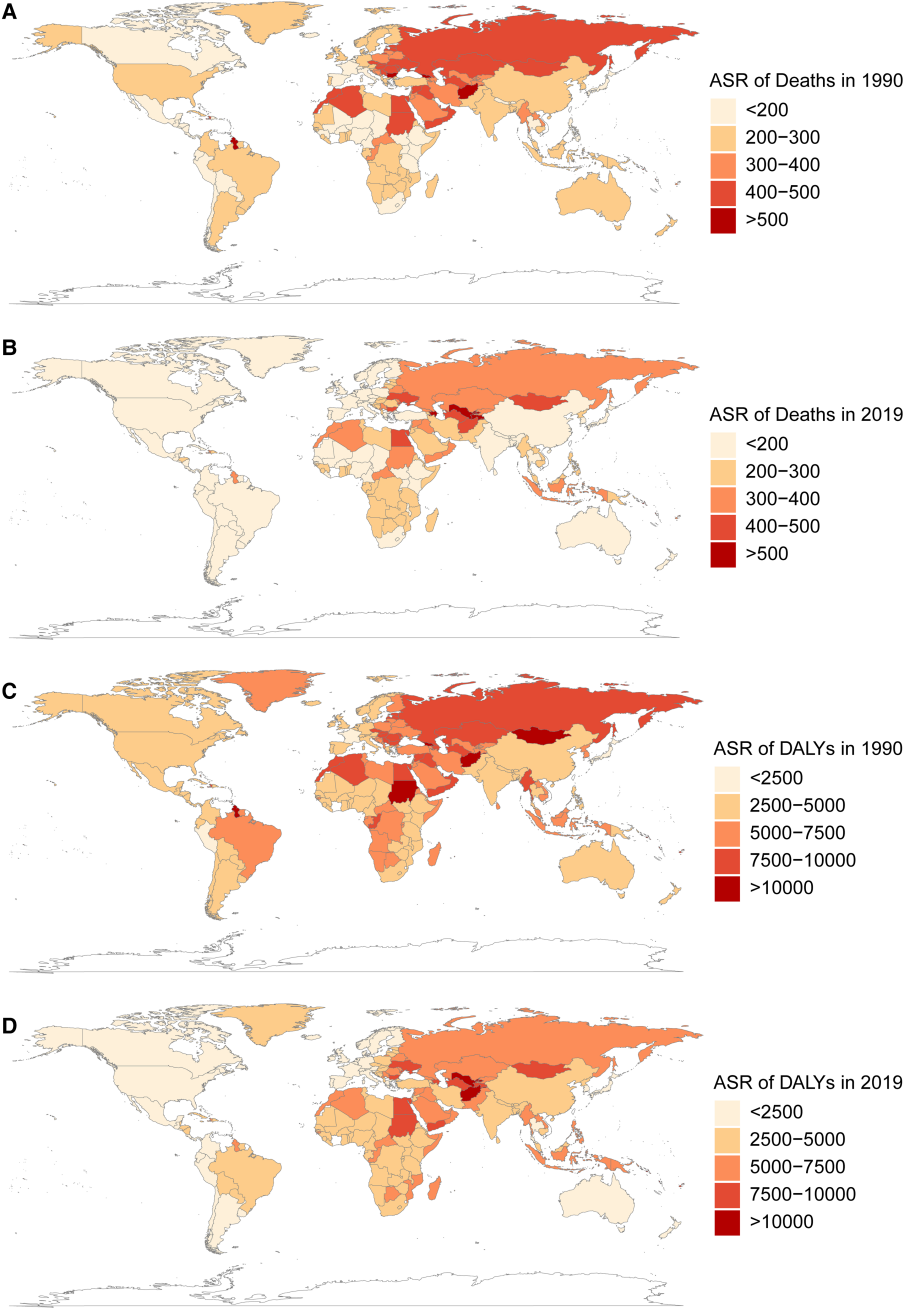
The global burden of metabolism-related CVDs in 204 countries and territories in 1990 and 2019. (**A,B**) the ASR (per 100,000 persons) of death attributable to metabolism-related CVDs in 1990 and 2019. (**C,D**) the ASR (per 100,000 persons) of DALYs attributable to metabolism-related CVDs in 1990 and 2019. CVDs, cardiovascular diseases; ASR, age standardized rate; DALYs, disability-adjusted life years.

From 1990 to 2019, the change in CVDs death and DALYs ASRs attributable to metabolic risk factors showed significant regional differences globally (Figure 3). The Eastern Mediterranean region had the greatest death and DALYs ASR in both 1990 and 2019, while the Americas had the lowest (Figure 3). The ASR of deaths has decreased by more than 100 per 100,000 in several regions (Central Europe, Australasia, Tropical Latin America, Western Europe, High-Income Asia Pacific), but many other regions (Central Latin America, East Asia, Andean Latin America, South Asia, Southeast Asia) had decreases in the death ASR of less than 50 per 100,000, and even increases were observed in Oceania and Central Asia.

**Figure 4:**
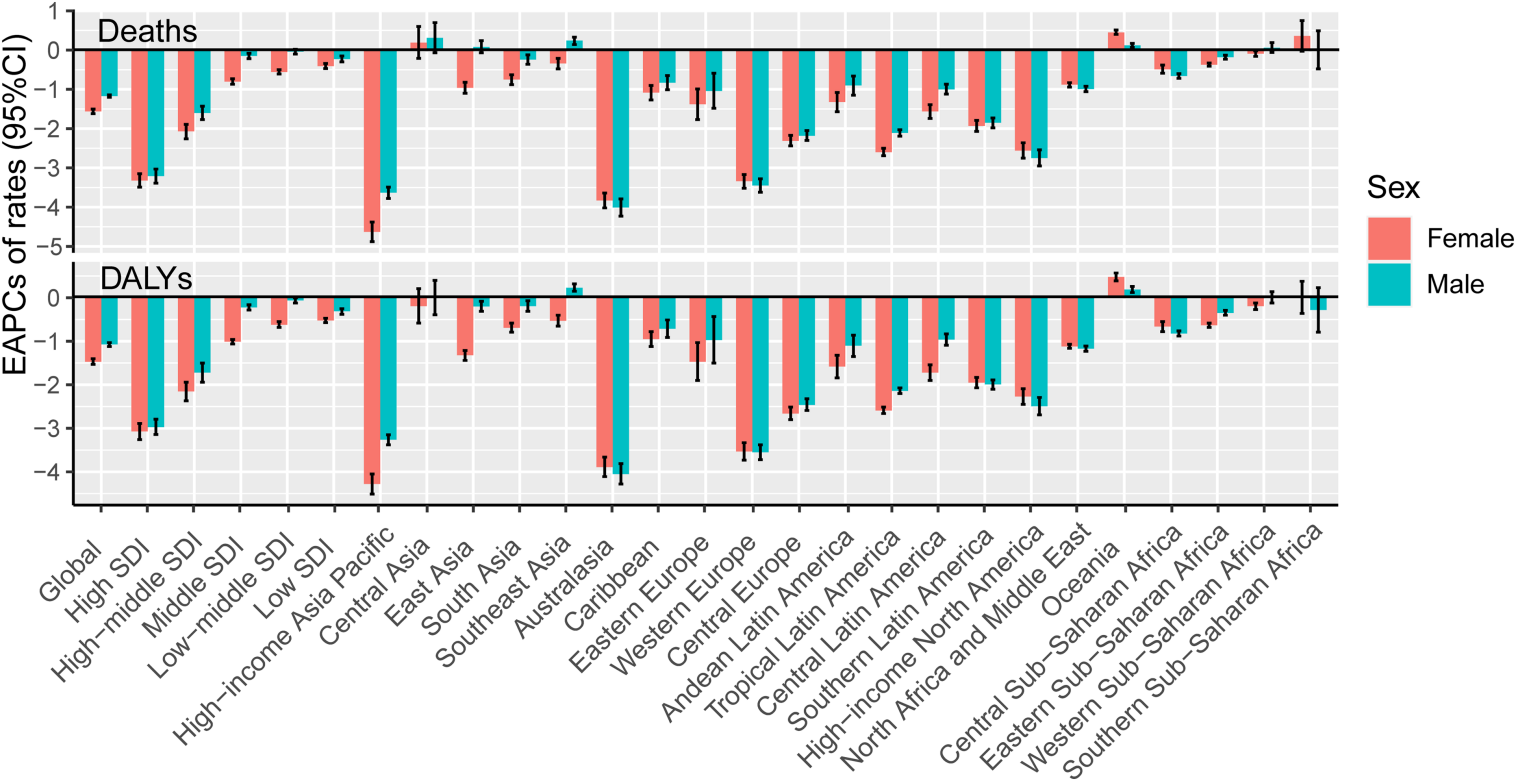
The annual variation in metabolism-related CVDs burden in young adults from 1990 to 2019, by location. EAPCs of Deaths/DALYs ASR (per 100 000 persons) in SDI quintiles and 21 GBD World Regions, by sex. EAPC, estimated annual percentage changes; SDI, social-demographic index; DALY, disability-adjusted life year.

According to the calculation of EAPCs, most regions had negative (declining) ASRs for deaths and DALYs (Figure 4). The area with the greatest reduction in deaths was High-income Asia Pacific [−4.0 (−4.2, −3.8)], while the area with the greatest reduction in DALYs was Australasia [−4.0 (−4.2, −3.7)].The three countries with the largest reductions of death ASRs were Bahrain, Czechia and Estonia. Uzbekistan, Tajikistan, and Azerbaijan had the largest increases. As for DALYS, the three countries with the largest decreases were Bahrain, Czechia and Mauritius, whereas Uzbekistan, Tajikistan and the Philippines had the largest increases (Supplementary Table S3).

### 3.4. Age and sex differences

**Figure 5:**
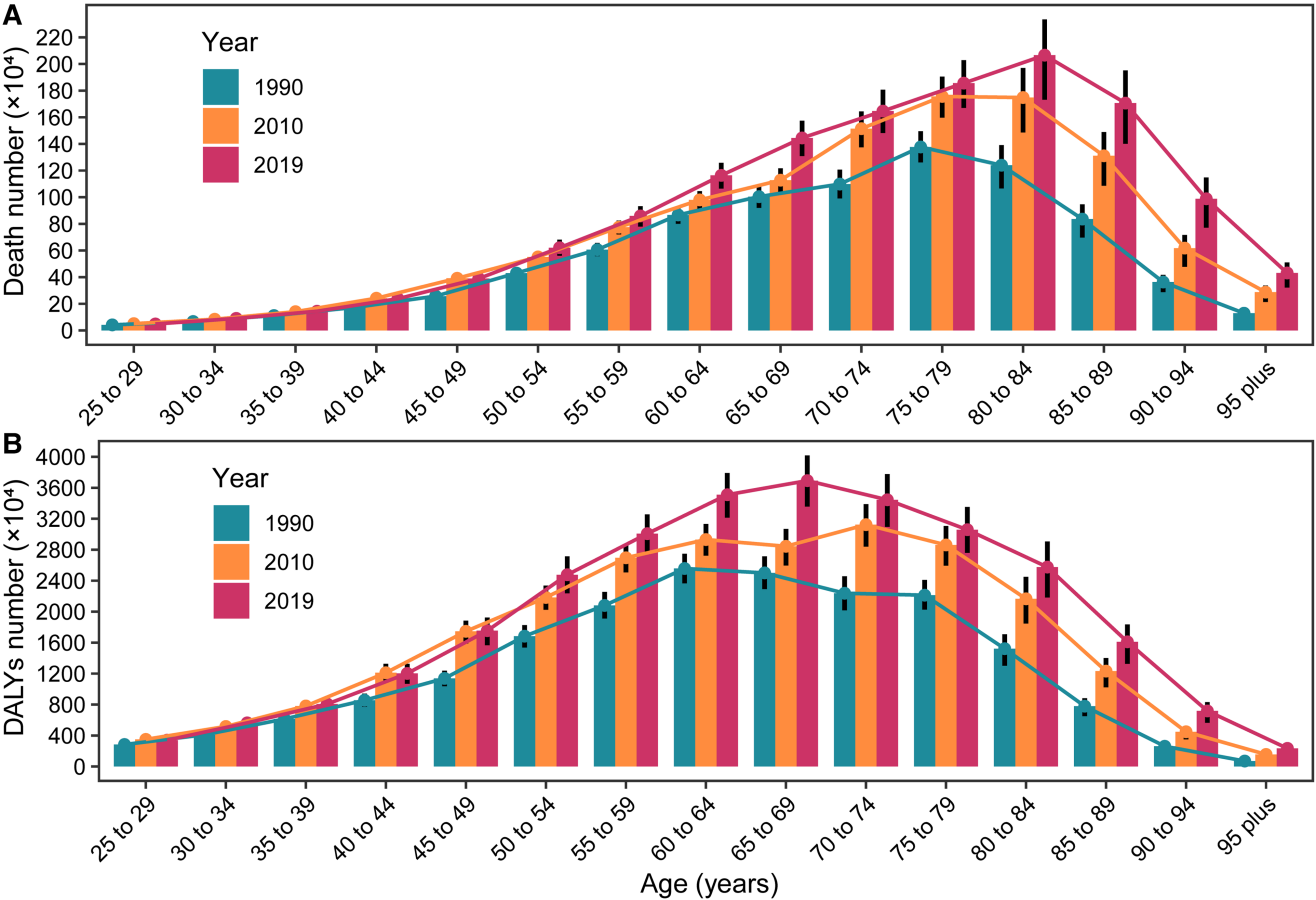
The global numbers deaths/DALYs in different patient age stratifications, in 1990, 2010 and 2019. (**A**) Death number in different age stratifications, in 1990, 2010 and 2019; (**B**) DALYs number in different age stratifications, in 1990, 2010 and 2019. DALYs, disability-adjusted life years.

The numbers of deaths and DALYs grew dramatically with increasing age. In 1990, the numbers of deaths and DALYs peaked in the 75–79 and 65–69 age groups, respectively. By 2010 and 2019, the peaks had shifted to the 80–84 and 70–74 age groups, respectively (Figure 5).

The burden of CVDs attributable to metabolic risk factors exhibited significant sex differences and changes over time. In 1990, there were more deaths number among women than men (4.34 vs. 4.27 million), a difference that lasted for five years, and in 1995, for the first time, there were more deaths among men than women (4.73 vs. 4.72 million), and this trend remained so until 2019. Since 1990, the number of DALYs and YLLs was consistently higher in men than in women, while the number of YLDs was lower than that in women. From 1990 to 2019, the ASRs for deaths, DALYs, and YLLs were consistently higher for men than for women, while YLDs were consistently lower.

### 3.5. Correlation between metabolic risk-related CVDs burden and socialdemographic index

**Figure 6:**
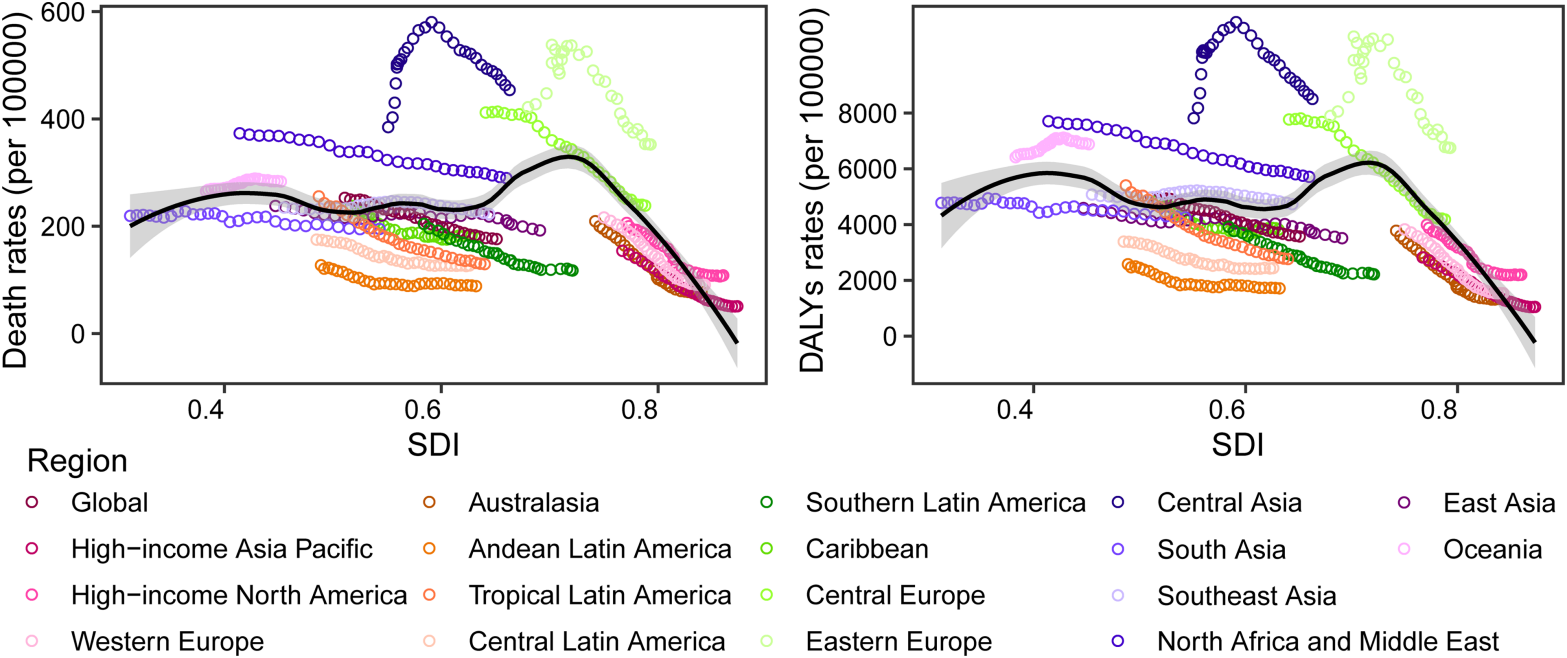
The trends in metabolism-related CVDs deaths/DALYs rates across 21 GBD world regions by SDI, 1990–2019. Each colored point line represents the rates for each year from 1990 to 2019 in a specified region. The black line shows estimate across the spectrum of the SDI. SDI, social-demographic index; DALY, disability-adjusted life year.

The age-standardized death and DALYs rates for CVDs attributable to metabolic factors varied widely with the SDI (Supplementary Table S4). As the SDI rose from 1990 to 2019 (0.5–0.7), the global ASR for deaths (from 252.9 to 176.1 per 100,000 persons) and DALYs (from 4,965.8 to 3,554.5 per 100,000 persons) fell steadily. Almost all regions, with the exception of Oceania and Central Asia, experienced a decline in the ASR for deaths and DALYs as the SDI rose. The intermediate SDI sector had the highest burden of CVDs, whereas the high SDI segment had the lowest burden, with both death and DALYs ASR significantly declining over the past several decades. (Supplementary Table S2 and Figure 6).

### 3.6. Prediction of the global burden of CVDs attributed to metabolic risks from 2020 to 2029

**Figure 7:**
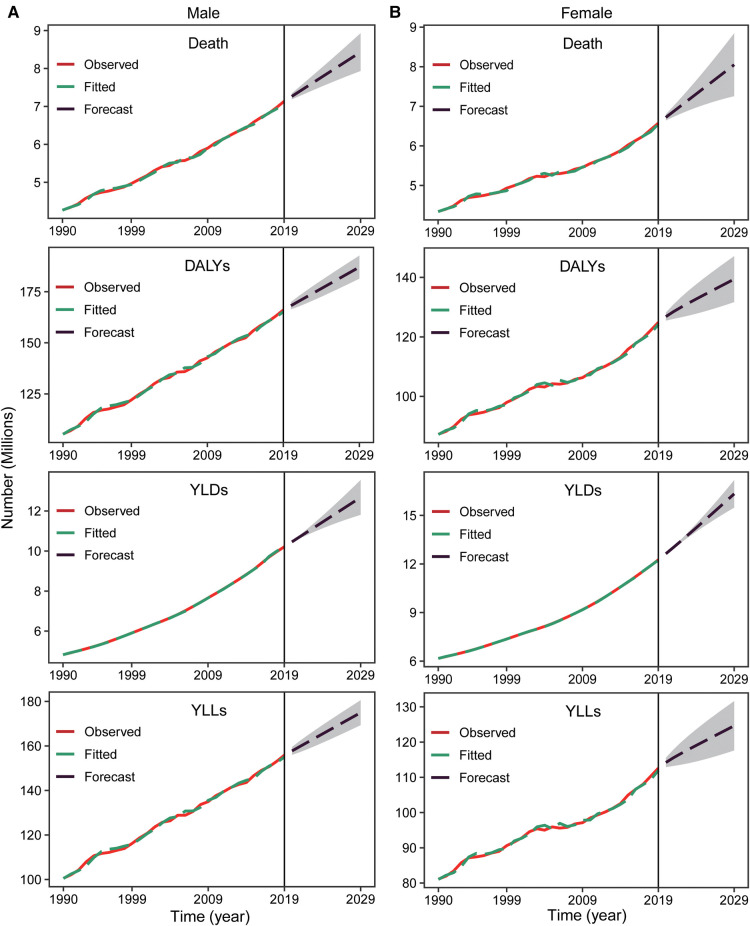
The prediction of metabolism-related CVDs deaths/DALYs numbers from 2020 to 2039. Number of CVDs deaths, DALYs, YLDs, and YLLs due to metabolic risk in male (**A**) and female (**B**). Shaded regions represent 95% uncertainty intervals. CVDs, cardiovascular diseases; DALYs, disability-adjusted life years; YLDs, years lived with disability; YLLs, years of life lost.

According to our predictions, the number of CVDs deaths, DALYs, YLDs and YLLs attributable to metabolic risk factors will continue to increase globally in the coming years for both men and women (Figure 7). The number of deaths will increase by 20.4%, from 13.7 million in 2019 to 16.5 million in 2029. The number of DALYs will also increase (11.7%) from 290.95 million to 324.8 million (Supplementary Table S5).

Similar to the past 30 years, the adjusted ASR of deaths, DALYs, YLDs, YLLs will gradually decrease globally except for female YLLs (Supplementary Figure S1). The ASR of deaths will drop from 176.1 per 100,000 in 2019 to 149.6 per 100,000 in 2029 (3,573.5–3,101.1 for DALYs, 3,298.6–2,827.5 for YLLs), but the ASR of YLDs will increase from 274.9 to 279.6 per 100,000 (Supplementary Table S5). There will be a clear gender difference in the change in YLDs; that for males will decrease from 265.6 to 261.0 per 100,000, but the change in YLDs for females will increase significantly (from 282.4 to 295.2 per 100,000).

## 4. Discussion

In this research, we systematically analyzed the global burden of CVDs attributable to metabolic risk factors and predicted future trends. From 1990 to 2019, the numbers of deaths, DALYs, YLDs, and YLLs attributable to CVDs increased significantly worldwide, and the ASR for all four indicators decreased significantly, and this trend will continue over the next 10 years. The number of deaths and DALYs are expected to reach 16.5 million and 324.8 million, respectively, by 2029. The global burden of CVDs attributed to metabolic factors also showed significant sex, geographic, and age differences. Metabolic risk factors have had the most important impact on and are highly connected with the outcomes of CVDs (2), and with the increase in the aging obese population and social pressures, the influence of metabolic factors may become even more important in the future.

Consistent with previous studies (20, 21), elevated SBP or hypertension is the greatest risk factor for disability and death from CVDs. The global prevalence of hypertension is increasing due to an aging population and increased lifestyle risk factors, including unhealthy diets and lack of physical activity. A total of 1.4 billion (31.1%) adults worldwide suffered from hypertension in 2010 (22), however, changes in the prevalence of hypertension are not uniform across the globe. Over the past two decades, the prevalence of hypertension has declined slightly in high-income countries, while it has increased significantly in low- and middle-income countries (23). Our analysis shows that 10.0 million CVDS deaths in 2019 can be attributed to elevated SBP, and we expect this number to continue to rise in the future as the number of people suffering from CVDs and hypertension increases.

As one of the earliest known risk factors, LDL-C lowering has been one of the cornerstones of CVDs therapy (24, 25). Our forecasts indicate that by 2029, the numbers of deaths and DALYs related to LDL-C elevation in patients with CVDs will reach 3.1 million and 63.2 million, respectively. Despite the availability of numerous LDL-C-lowering medications, LDL-C compliance rates remain inadequate in some regions, particularly in developing nations (26). In 2016, the rates of dyslipidemia awareness, treatment, and control among Chinese adults were 31.0%, 19.5%, and 8.9%, respectively (27). Obesity is not only a risk factor for CVDs, but also a factor that can influence or aggravate other risk factors such as hypertension, dyslipidemia and diabetes through various mechanisms (28–31). The global incidence of obesity has risen dramatically over the past 50 years, with an average prevalence of 19.5 percent among adults worldwide in 2015. CVDs are the leading cause of disability and death in patients with elevated FBG or diabetes. Diabetes mellitus affects approximately 1 in 11 adults worldwide (90 percent is type 2 diabetes mellitus) (32). The incidence of diabetes is rising globally, with the highest increases occurring in low- and middle-income nations. Type 2 diabetes is quickly surpassing communicable diseases as the main cause of kidney damage in countries with weaker economies and is competing for limited healthcare resources (33). Hypertension, diabetes, obesity, dyslipidemia, and abnormal renal function are not only metabolic risk factors for CVDs, but also factors that influence and promote each other and further impact on CVDs. Therefore, more effective management of these diseases is important to reduce the global burden of CVDs.

The burden of CVDs attributable to metabolic risk factors is significantly higher in men than in women, both for deaths and DALYs, which may be related to the protective effect of hormones in women and to the higher prevalence of these risk factors in men (34–37). We also discovered a significant shift in the peak incidence of death and disability in patients with CVDs attributed to metabolic risk variables at various ages over the past three decades, which is consistent with the global trend of aging in many countries. In 2022, there were 771 million individuals aged 65 or older worldwide, three times as many as in 1980 (258 million). It is anticipated that the senior population will reach 994 million by 2030 and 1.6 billion by 2050 (38). Considering that older persons often have several risk factors, a greater incidence of CVDs, and a higher mortality rate (39–41), the global burden of CVDs will increase substantially as the global population ages.

The SDI reflects socioeconomic development. The burden of CVDs associated with metabolic risk factors decreases progressively with increasing SDI, with the most pronounced reductions in countries with a high SDI, but it is important to note that although the data analysis suggests that the burden of disease is lower in areas with a low SDI than in areas with an intermediate SDI, this may be related to the underestimation of the burden of CVDs resulting from the poor health care system and lack of medical resources in low-SDI regions.

Our research has the following limitations: first, because the GBD database included only the metabolic risk factors analyzed in this study, we did not include risk factors such as elevated homocysteine and decreased high-density lipoprotein cholesterol (HDL-C) (42, 43); second, the majority of the studies included in the GBD analysis represent primarily North American and European populations. In certain developing nations, particularly in regions with a low SDI, data are scant and limited, leading to biased outcomes. Third, when projecting the future burden of CVDs, we did not account for future changes in population size, which may have led to biased forecasts.

## 5. Conclusions

The burden of CVDs attributable to metabolic risk factors increased from 1990 to 2019 with significant regional, sex, and age differences, and this trend is likely to continue. The aging of the population and the increasing incidence of metabolic diseases may worsen this trend. More focus should be placed on metabolic disorders to reduce the burden of CVDs.

## Data Availability

The original contributions presented in the study are included in the article/**Supplementary Material**, further inquiries can be directed to the corresponding authors.
