# Supplementary material for: The burden of cardiovascular diseases attributable to metabolic risk factors and its change from 1990 to 2019: a systematic analysis and prediction
Source: Front Epidemiol. 2023 May 25;3:1048515. doi: 10.3389/fepid.2023.1048515 (PMC10910969; doi:10.3389/fepid.2023.1048515)
Supplement: Supplementary file 1 [file Datasheet1.docx]

**The burden of cardiovascular diseases attributable to metabolic risk factors and its change from 1990 to 2019: A systematic analysis and prediction**

Huaigen Wang ^1^, Jing Liu^1^, Yunfei Feng^1^, Aiqun Ma^1,2,3*^, Tingzhong Wang ^1,2,3*^

1. Department of Cardiovascular Medicine, The First Affiliated Hospital of Xi'an Jiaotong University, Xi'an, Shaanxi, P. R. China

2. Key Laboratory of Molecular Cardiology, Shaanxi Province, P. R. China

3. Key Laboratory of Environment and Genes Related to Diseases, Xi'an Jiaotong University, Ministry of Education, Xi'an, Shaanxi, P. R. China

* Correspondence:

Aiqun Ma

aiqun.ma@xjtu.edu.cn

Tingzhong Wang

tingzhong.wang@xjtu.edu.cn

**Supplement Materials**

Contents

**Figure S1 The prediction of metabolism-related CVDs deaths/DALYs ASR from 2020 to 20393**

**Table S1 The proportion of each CVDs attributed to individual metabolic risk factors in 20194**

**Table S2 The EAPCs of deaths/DALYs rates (per 100 000 persons) in SDI quintiles and 21 GBD World Regions, by sex5**

**Table S3 The deaths and DALYs ASR of CVDs attributed to metabolic risk factors in 204 locations in 1990 and 201910**

**Table S4 Metabolism-related CVDs deaths/DALYs ASR across 21 GBD world regions and SDI in 1990 and 201912**

**Table S5 Number and ASR of metabolism-related CVDs deaths/DALYs/YLDs/YLLs from 1990 to 202914**


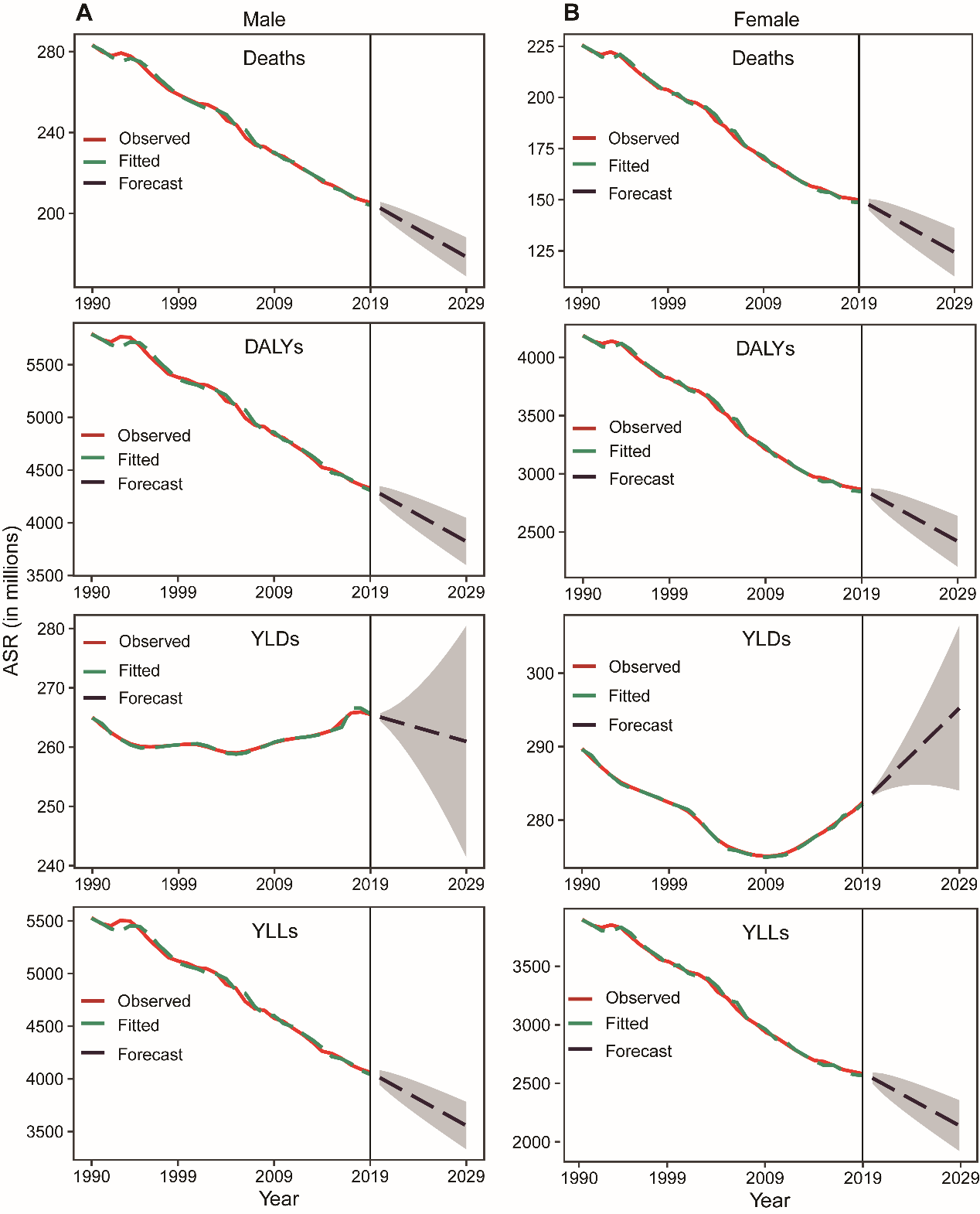


**Figure S1** **The prediction of metabolism-related CVDs deaths/DALYs ASR from 2020 to 2039.** ASR of CVDs deaths, DALYs, YLDs, and YLLs due to metabolic risk in male(A) and female(B). Shaded regions represent 95% uncertainty intervals. CVDs, cardiovascular diseases; DALYs, disability-adjusted life years; YLDs, years lived with disability; YLLs, years of life lost.

Table S1 The proportion of each CVDs attributed to individual metabolic risk factors in 2019

| Cardiovascular diseases | metabolic risk factors | deaths | | DALYs | |
| --- | --- | --- | --- | --- | --- |
|  |  | number | percent(%) | number | percent(%) |
| Total | Kidney dysfunction | 1734320 | 7.5 | 34719406 | 6.9 |
|  | high LDL-C | 4396983 | 19.0 | 98618021 | 19.5 |
|  | high FBG | 3763298 | 16.3 | 72591163 | 14.3 |
|  | high SBP | 9965974 | 43.2 | 213915370 | 42.2 |
|  | high BMI | 3226966 | 14.0 | 86714628 | 17.1 |
| Rheumatic heart disease | high SBP | 76408 | 100.0 | 2446377 | 100.0 |
| Stroke | Kidney dysfunction | 504061 | 7.2 | 28908396 | 17.1 |
|  | high LDL-C | 612650 | 8.7 | 79556713 | 47.1 |
|  | high FBG | 1389769 | 19.7 | 34871340 | 20.6 |
|  | high SBP | 3444322 | 48.9 | 11949056 | 7.1 |
|  | high BMI | 1090642 | 15.5 | 13701627 | 8.1 |
| Ischemic heart disease | Kidney dysfunction | 1220783 | 8.8 | 43253265 | 14.8 |
|  | high LDL-C | 3784333 | 27.3 | 99397294 | 34.1 |
|  | high FBG | 2353422 | 17.0 | 41369773 | 14.2 |
|  | high SBP | 4861616 | 35.0 | 22516856 | 7.7 |
|  | high BMI | 1662339 | 12.0 | 84916394 | 29.1 |
| Hypertensive heart disease | high BMI | 410145 | 26.2 | 21508002 | 71.2 |
|  | high SBP | 1156733 | 73.8 | 8704832 | 28.8 |
| Non-rheumatic valvular heart disease | high SBP | 39749 | 100.0 | 628311 | 100.0 |
| Cardiomyopathy and myocarditis | high SBP | 82170 | 100.0 | 1970867 | 100.0 |
| Atrial fibrillation and flutter | high SBP | 107091 | 62.7 | 3337876 | 65.4 |
|  | high BMI | 63841 | 37.4 | 1768682 | 34.6 |
| Aortic aneurysm | high SBP | 59880 | 100.0 | 1241450 | 100.0 |
| Peripheral artery disease | high FBG | 20107 | 41.4 | 429501 | 38.8 |
|  | high SBP | 19019 | 39.1 | 424394 | 38.3 |
|  | Kidney dysfunction | 9476 | 19.5 | 253494 | 22.9 |
| Endocarditis | high SBP | 21863 | 100.0 | 544406 | 100.0 |
| Other cardiovascular and circulatory diseases | high SBP | 97121 | 100.0 | 2859680 | 100.0 |
| DALYs, Disability-Adjusted Life Years; LDL-C, low-density lipoprotein cholesterol; FBG, fasting plasma glucose; SBP, systolic blood pressure; BMI, high body-mass index. | | | | | |

Table S2 The EAPCs of deaths/DALYs rates (per 100 000 persons) in SDI quintiles and 21 GBD World Regions, by sex.

| location | deaths | | | DALYs | | |
| --- | --- | --- | --- | --- | --- | --- |
|  | both | male | female | both | male | female |
| Global | -1.4 (-1.4-1.3) | -1.2 (-1.2-1.1) | -1.6(-1.6-1.5) | -1.2 (-1.3-1.2) | -1.1 (-1.1-1.0) | -1.5 (-1.5-1.4) |
| High SDI | -3.2 (-3.4-3.0) | -3.2 (-3.4-3.0) | -3.3 (-3.5-3.2) | -2.9 (-3.-2.8) | -3.0 (-3.1-2.8) | -3.1 (-3.3-2.9) |
| High-middle SDI | -1.8 (-2.0-1.7) | -1.6 (-1.8-1.4) | -2.1 (-2.3-1.9) | -1.9 (-2.1-1.7) | -1.7 (-1.9-1.5) | -2.2 (-2.4-1.9) |
| Middle SDI | -0.5 (-0.5-0.4) | -0.2 (-0.2-0.1) | -0.8 (-0.9-0.7) | -0.6 (-0.6-0.5) | -0.2 (-0.3-0.2) | -1.0 (-1.1-1.0) |
| Low-middle SDI | -0.3 (-0.4-0.3) | -0.04 (-0.10-0.04) | -0.6 (-0.6-0.5) | -0.3 (-0.4-0.3) | -0.06 (-0.12-0.01) | -0.6 (-0.7-0.6) |
| Low SDI | -0.3 (-0.4-0.3) | -0.2 (-0.3-0.2) | -0.4 (-0.5-0.3) | -0.4 (-0.5-0.4) | -0.3 (-0.4-0.3) | -0.5 (-0.6-0.5) |
| High-income Asia Pacific | -4.0 (-4.2-3.8) | -3.6 (-3.8-3.5) | -4.6 (-4.9-4.4) | -3.6 (-3.8-3.4) | -3.3(-3.4-3.2) | -4.3 (-4.5-4.1) |
| Central Asia | 0.3 (-0.1-0.7) | 0.3 (-0.1-0.7) | 0.2 (-0.2-0.6) | -0.07 (-0.47-0.33) | 0 (-0.39-0.4) | -0.2 (-0.6-0.2) |
| East Asia | -0.5 (-0.6-0.3) | 0.1 (-0.1-0.2) | -1.0 (-1.1-0.8) | -0.7 (-0.8-0.6) | -0.2 (-0.3-0.1) | -1.3 (-1.4-1.2) |
| South Asia | -0.5 (-0.6-0.4) | -0.2 (-0.4-0.1) | -0.8 (-0.9-0.6) | -0.4 (-0.5-0.3) | -0.2 (-0.3-0.1) | -0.7 (-0.8-0.6) |
| Southeast Asia | -0.1 (-0.2-0.1) | 0.2 (0.1-0.3) | -0.3 (-0.5-0.2) | -0.11 (-0.21-0.01) | 0.2 (0.2-0.3) | -0.5 (-0.7-0.4) |
| Australasia | -3.9 (-4.1-3.7) | -4.0 (-4.2-3.8) | -3.8 (-4.0-3.6) | -4.0 (-4.2-3.7) | -4.1 (-4.3-3.8) | -3.9 (-4.1-3.7) |
| Caribbean | -0.1 (-1.1-0.8) | -0.8 (-1.0-0.7) | -1.1 (-1.3-0.9) | -0.8 (-1.0-0.6) | -0.7 (-0.9-0.5) | -1.0 (-1.1-0.8) |
| Eastern Europe | -1.2 (-1.6-0.7) | -1.0 (-1.5-0.6) | -1.4 (-1.8-1.0) | -1.2 (-1.7-0.7) | -1.0 (-1.5-0.4) | -1.5 (-1.9-1.0) |
| Western Europe | -3.3 (-3.5-3.2) | -3.5 (-3.6-3.3) | -3.3 (-3.5-3.2) | -3.5 (-3.7-3.3) | -3.6 (-3.7-3.4) | -3.5 (-3.7-3.3) |
| Central Europe | -2.2 (-2.4-2.1) | -2.2 (-2.3-2.1) | -2.3 (-2.4-2.2) | -2.5 (-2.7-2.4) | -2.5 (-2.6-2.3) | -2.7 (-2.8-2.5) |
| Andean Latin America | -1.1 (-1.4-0.9) | -0.9 (-1.6-0.7) | -1.3 (-1.6-1.1) | -1.3 (-1.6-1.1) | -1.1 (-1.4-0.9) | -1.6 (-1.8-1.3) |
| Tropical Latin America | -2.4 (-2.5-2.3) | -2.1 (-2.2-2.0) | -2.6 (-2.7-2.5) | -2.4 (-2.4-2.3) | -2.1 (-2.2-2.1) | -2.6 (-2.7-2.5) |
| Central Latin America | -1.3 (-1.4-1.1) | -1.0 (-1.1-0.9) | -1.6 (-1.7-1.4) | -1.3 (-1.5-1.8) | -1.0 (-1.1-0.8) | -1.7 (-1.9-1.5) |
| Southern Latin America | -19 (-2.029-1.8) | -1.9 (-2.0-1.7) | -1.9 (-2.1-1.8) | -2.0 (-2.1-1.9) | -2.0 (-2.1-1.9) | -2.0 (-2.1-1.8) |
| High-income North America | -2.6 (-2.8-2.4) | -2.8 (-3.0-2.5) | -2.6 (-2.8-2.4) | -2.4 (-2.5-2.2) | -2.5 (-2.7-2.3) | -2.3 (-2.5-2.1) |
| North Africa and Middle East | -0.9 (-1.0-0.9) | -1.0 (-1.1-0.9) | -0.9 (-0.9-0.8) | -1.2 (-1.2-1.1) | -1.2 (-1.2-1.1) | -1.1 (-1.2-1.1) |
| Oceania | 0.3 (0.2-0.3) | 0.1 (0.1-0.2) | 0.5 (0.4-0.5) | 0.3 (0.2-0.4) | 0.2 (0.1-0.3) | 0.5 (0.4-0.6) |
| Central Sub-Saharan Africa | -0.5 (-0.6-0.4) | -0.7 (-0.7-0.6) | -0.5 (-0.6-0.4) | -0.7 (-0.8-0.6) | -0.8 (-0.9-0.8) | -0.7 (-0.8-0.6) |
| Eastern Sub-Saharan Africa | -0.3 (-0.3-0.3) | -0.2 (-0.2-0.1) | -0.4 (-0.4-0.3) | -0.5 (-0.5-0.5) | -0.4 (-0.4-0.3) | -0.6 (-0.7-0.6) |
| Western Sub-Saharan Africa | -0.1 (-0.1-0.1) | 0.1 (-0.1-0.2) | -0.09 (-0.16-0.02) | -0.12 (-0.22-0.02) | 0.01 (-0.12-0.14) | -0.2 (-0.3-0.1) |
| Southern Sub-Saharan Africa | 0.2 (-0.2-0.6) | 0 (-0.5-0.5) | 0.4 (-0.03-0.8) | -0.1 (-0.6-0.3) | -0.3 (-0.8-0.2) | 0.01 (-0.36-0.38) |
| EAPC, estimated annual percentage changes; DALYs, Disability-Adjusted Life Years; SDI, social-demographic index | | | | | | |

Table S3 The deaths and DALYs ASR of CVDs attributed to metabolic risk factors in 204 locations in 1990 and 2019

|  | ASR of deaths | | | ASR of DALYs | | |
| --- | --- | --- | --- | --- | --- | --- |
| location | 1990 | 2019 | change | 1990 | 2019 | change |
| Bahrain | 478.8 | 191.8 | -286.9 | 9172.1 | 3309.8 | -5862.2 |
| Czechia | 422.1 | 177.0 | -245.1 | 7986.7 | 2981.1 | -5005.6 |
| Mauritius | 376.7 | 167.4 | -209.2 | 8284.2 | 3511.4 | -4772.8 |
| Hungary | 439.6 | 233.2 | -206.4 | 8605.3 | 4148.6 | -4456.7 |
| Estonia | 438.5 | 220.5 | -218.0 | 8166.0 | 3722.0 | -4444.0 |
| Saint Kitts and Nevis | 435.0 | 219.8 | -215.3 | 8591.0 | 4183.0 | -4408.1 |
| Maldives | 328.8 | 156.4 | -172.4 | 7171.5 | 2982.0 | -4189.5 |
| Poland | 369.9 | 174.5 | -195.4 | 7253.6 | 3138.7 | -4114.9 |
| Republic of Korea | 268.3 | 61.7 | -206.7 | 5045.7 | 1069.7 | -3976.0 |
| Slovakia | 414.2 | 237.4 | -176.9 | 8057.1 | 4082.6 | -3974.5 |
| Guyana | 511.6 | 342.0 | -169.6 | 11089.0 | 7250.2 | -3838.8 |
| Qatar | 428.9 | 273.3 | -155.6 | 7841.8 | 4124.1 | -3717.7 |
| Algeria | 498.6 | 317.8 | -180.8 | 8836.4 | 5268.8 | -3567.6 |
| Croatia | 385.9 | 209.5 | -176.4 | 6819.4 | 3390.2 | -3429.2 |
| Latvia | 433.1 | 272.3 | -160.8 | 8278.9 | 4873.1 | -3405.7 |
| United Arab Emirates | 455.6 | 271.6 | -183.9 | 8832.6 | 5500.9 | -3331.6 |
| Ireland | 267.6 | 96.0 | -171.6 | 4906.8 | 1597.5 | -3309.3 |
| Georgia | 529.3 | 364.0 | -165.3 | 10223.6 | 6960.6 | -3262.9 |
| Finland | 283.6 | 130.5 | -153.0 | 5333.3 | 2136.9 | -3196.5 |
| Bermuda | 268.8 | 104.8 | -164.0 | 5049.8 | 1893.0 | -3156.7 |
| Jordan | 356.7 | 205.9 | -150.9 | 7024.1 | 3907.4 | -3116.7 |
| Romania | 467.9 | 295.2 | -172.7 | 8316.3 | 5235.9 | -3080.4 |
| Trinidad and Tobago | 335.0 | 179.8 | -155.2 | 6755.6 | 3713.5 | -3042.0 |
| Brunei Darussalam | 340.4 | 196.3 | -144.1 | 6626.9 | 3599.5 | -3027.4 |
| United Kingdom | 252.5 | 91.6 | -160.9 | 4687.6 | 1660.6 | -3027.0 |
| Denmark | 245.4 | 83.2 | -162.2 | 4409.6 | 1402.6 | -3007.0 |
| Germany | 279.0 | 119.8 | -159.3 | 4872.4 | 1930.8 | -2941.6 |
| Singapore | 202.0 | 66.5 | -135.4 | 4187.3 | 1332.3 | -2855.0 |
| Slovenia | 257.9 | 109.6 | -148.4 | 4733.7 | 1893.9 | -2839.8 |
| Portugal | 253.4 | 89.2 | -164.2 | 4369.8 | 1530.5 | -2839.3 |
| Greenland | 281.8 | 147.3 | -134.5 | 5813.0 | 2973.9 | -2839.1 |
| Norway | 224.6 | 78.6 | -146.0 | 4194.4 | 1364.2 | -2830.2 |
| Equatorial Guinea | 298.1 | 204.5 | -93.6 | 6696.3 | 3874.1 | -2822.2 |
| Cyprus | 316.2 | 139.3 | -176.8 | 5010.7 | 2189.2 | -2821.6 |
| Oman | 474.6 | 398.4 | -76.3 | 9608.8 | 6837.5 | -2771.3 |
| Iran (Islamic Republic of) | 344.3 | 218.7 | -125.6 | 6733.8 | 3999.4 | -2734.4 |
| Turkey | 279.8 | 169.6 | -110.2 | 5840.1 | 3107.6 | -2732.5 |
| Malta | 261.1 | 113.0 | -148.1 | 4645.6 | 1940.6 | -2705.1 |
| Brazil | 258.0 | 129.1 | -129.0 | 5452.0 | 2761.5 | -2690.5 |
| Luxembourg | 229.8 | 83.1 | -146.7 | 4084.0 | 1426.2 | -2657.9 |
| New Zealand | 222.6 | 94.5 | -128.2 | 4223.0 | 1611.1 | -2611.9 |
| Saint Lucia | 301.8 | 155.2 | -146.6 | 5561.4 | 2970.5 | -2590.8 |
| Israel | 210.8 | 69.0 | -141.9 | 3761.8 | 1187.6 | -2574.2 |
| Rwanda | 264.2 | 172.8 | -91.3 | 5897.0 | 3348.1 | -2548.9 |
| Serbia | 446.8 | 353.8 | -93.1 | 8074.6 | 5534.5 | -2540.1 |
| Lithuania | 378.6 | 257.3 | -121.4 | 7094.4 | 4582.7 | -2511.8 |
| Australia | 206.8 | 73.6 | -133.2 | 3689.4 | 1279.8 | -2409.7 |
| Myanmar | 339.3 | 262.0 | -77.3 | 7678.9 | 5318.4 | -2360.5 |
| Grenada | 303.9 | 196.3 | -107.6 | 6118.3 | 3864.5 | -2253.8 |
| Sudan | 465.5 | 389.6 | -76.0 | 10010.2 | 7795.2 | -2215.0 |
| Seychelles | 291.4 | 200.5 | -90.9 | 6388.4 | 4207.8 | -2180.6 |
| Austria | 225.7 | 111.5 | -114.2 | 3951.8 | 1783.1 | -2168.7 |
| Lebanon | 367.6 | 270.2 | -97.4 | 7386.9 | 5236.5 | -2150.4 |
| Congo | 352.0 | 278.3 | -73.6 | 7720.2 | 5584.7 | -2135.4 |
| Colombia | 205.2 | 98.4 | -106.8 | 4011.9 | 1878.8 | -2133.1 |
| Afghanistan | 530.9 | 464.8 | -66.1 | 12201.2 | 10086.4 | -2114.8 |
| Sweden | 213.3 | 99.2 | -114.1 | 3775.0 | 1666.8 | -2108.2 |
| Republic of Moldova | 452.2 | 325.1 | -127.1 | 8043.7 | 5969.6 | -2074.2 |
| Netherlands | 179.9 | 75.0 | -104.9 | 3341.9 | 1288.1 | -2053.8 |
| Palestine | 384.5 | 302.1 | -82.4 | 7549.6 | 5542.3 | -2007.3 |
| Taiwan (Province of China) | 178.8 | 72.5 | -106.3 | 3536.4 | 1574.3 | -1962.1 |
| Bulgaria | 523.6 | 424.2 | -99.4 | 9574.4 | 7616.6 | -1957.8 |
| Iceland | 183.0 | 80.0 | -103.0 | 3317.3 | 1374.0 | -1943.3 |
| Cook Islands | 288.7 | 205.6 | -83.1 | 6653.3 | 4733.6 | -1919.8 |
| Italy | 196.7 | 88.9 | -107.8 | 3328.0 | 1420.4 | -1907.6 |
| Russian Federation | 428.6 | 326.8 | -101.9 | 8149.2 | 6303.3 | -1845.9 |
| Canada | 171.8 | 74.6 | -97.2 | 3248.6 | 1405.3 | -1843.3 |
| Kuwait | 241.5 | 153.5 | -88.0 | 5040.4 | 3203.9 | -1836.5 |
| Mongolia | 488.1 | 425.2 | -62.9 | 10420.8 | 8594.4 | -1826.4 |
| Sri Lanka | 253.5 | 166.2 | -87.3 | 5035.9 | 3232.0 | -1803.9 |
| Uruguay | 202.1 | 111.4 | -90.8 | 3938.6 | 2137.3 | -1801.3 |
| Haiti | 406.9 | 342.2 | -64.7 | 8986.2 | 7194.6 | -1791.6 |
| Bosnia and Herzegovina | 329.4 | 260.8 | -68.5 | 6255.9 | 4465.1 | -1790.8 |
| United States of America | 209.6 | 112.6 | -97.0 | 4070.8 | 2294.3 | -1776.5 |
| Belgium | 176.6 | 81.5 | -95.1 | 3174.3 | 1427.9 | -1746.4 |
| Spain | 177.3 | 73.2 | -104.1 | 3024.0 | 1282.8 | -1741.2 |
| Mauritania | 229.6 | 163.3 | -66.4 | 4958.1 | 3224.2 | -1733.9 |
| Chile | 197.1 | 98.9 | -98.2 | 3540.2 | 1814.5 | -1725.8 |
| Puerto Rico | 180.2 | 83.8 | -96.4 | 3444.0 | 1727.3 | -1716.7 |
| Switzerland | 165.3 | 74.3 | -91.0 | 2840.1 | 1126.8 | -1713.3 |
| Armenia | 348.2 | 267.8 | -80.5 | 6436.9 | 4752.8 | -1684.1 |
| Syrian Arab Republic | 431.4 | 396.1 | -35.4 | 9128.0 | 7452.9 | -1675.2 |
| Kiribati | 432.4 | 395.8 | -36.6 | 12249.4 | 10583.6 | -1665.8 |
| Argentina | 207.8 | 126.5 | -81.3 | 4062.9 | 2407.6 | -1655.3 |
| Greece | 233.8 | 132.8 | -101.0 | 3963.7 | 2348.4 | -1615.3 |
| Iraq | 412.8 | 364.1 | -48.7 | 8782.4 | 7195.5 | -1586.9 |
| Cuba | 218.9 | 141.9 | -77.0 | 4216.9 | 2678.7 | -1538.1 |
| Burundi | 256.8 | 208.0 | -48.8 | 5815.1 | 4336.9 | -1478.2 |
| Fiji | 391.6 | 345.3 | -46.2 | 9393.5 | 7917.9 | -1475.6 |
| Ethiopia | 184.0 | 136.6 | -47.4 | 4205.4 | 2744.1 | -1461.4 |
| North Macedonia | 466.5 | 441.1 | -25.4 | 8512.6 | 7060.7 | -1451.9 |
| Monaco | 178.1 | 97.6 | -80.5 | 3111.1 | 1684.7 | -1426.5 |
| Malaysia | 263.5 | 208.3 | -55.2 | 5839.7 | 4488.3 | -1351.4 |
| Japan | 137.0 | 49.6 | -87.5 | 2408.3 | 1062.7 | -1345.5 |
| Venezuela (Bolivarian Republic of) | 242.7 | 181.4 | -61.4 | 4969.7 | 3624.5 | -1345.2 |
| Suriname | 258.3 | 187.9 | -70.5 | 5412.3 | 4068.3 | -1344.0 |
| Yemen | 432.7 | 391.5 | -41.2 | 9287.0 | 7948.9 | -1338.1 |
| Antigua and Barbuda | 220.0 | 167.7 | -52.3 | 4360.5 | 3066.3 | -1294.2 |
| France | 132.5 | 60.0 | -72.6 | 2377.9 | 1083.9 | -1294.0 |
| Albania | 285.9 | 228.5 | -57.3 | 5182.1 | 3945.4 | -1236.7 |
| Saint Vincent and the Grenadines | 281.3 | 217.2 | -64.2 | 5263.6 | 4065.4 | -1198.2 |
| Costa Rica | 169.6 | 102.5 | -67.2 | 3196.8 | 2007.1 | -1189.7 |
| Angola | 265.2 | 230.8 | -34.4 | 5868.8 | 4696.1 | -1172.6 |
| Kazakhstan | 407.1 | 386.1 | -21.0 | 8170.2 | 7009.9 | -1160.3 |
| Barbados | 198.5 | 134.9 | -63.5 | 3705.6 | 2547.9 | -1157.7 |
| Panama | 155.9 | 93.6 | -62.4 | 2936.4 | 1809.6 | -1126.9 |
| Dominica | 250.5 | 190.4 | -60.1 | 4699.6 | 3590.2 | -1109.4 |
| Lao People's Democratic Republic | 306.5 | 281.9 | -24.6 | 6954.5 | 5846.8 | -1107.7 |
| Thailand | 138.6 | 82.7 | -56.0 | 2970.0 | 1893.9 | -1076.1 |
| China | 239.4 | 195.1 | -44.3 | 4601.1 | 3535.8 | -1065.3 |
| Gabon | 270.3 | 233.3 | -37.0 | 5631.2 | 4573.4 | -1057.8 |
| Bahamas | 243.2 | 191.7 | -51.5 | 5090.1 | 4040.2 | -1050.0 |
| Peru | 111.2 | 63.3 | -48.0 | 2255.0 | 1247.9 | -1007.2 |
| El Salvador | 156.8 | 120.4 | -36.4 | 3358.6 | 2355.1 | -1003.5 |
| Guatemala | 171.2 | 131.1 | -40.1 | 3331.5 | 2361.4 | -970.1 |
| Tunisia | 297.8 | 253.0 | -44.8 | 5544.1 | 4602.0 | -942.1 |
| Niue | 333.1 | 298.4 | -34.7 | 7750.3 | 6815.8 | -934.5 |
| Cambodia | 231.1 | 214.5 | -16.5 | 5189.5 | 4263.7 | -925.8 |
| Morocco | 401.0 | 384.8 | -16.3 | 8212.9 | 7296.3 | -916.6 |
| Comoros | 227.4 | 194.7 | -32.8 | 4864.5 | 3949.3 | -915.3 |
| Bolivia (Plurinational State of) | 180.1 | 148.9 | -31.2 | 3604.6 | 2704.5 | -900.1 |
| Namibia | 263.4 | 232.6 | -30.7 | 5345.0 | 4473.8 | -871.2 |
| Democratic Republic of the Congo | 264.7 | 235.8 | -28.9 | 5582.8 | 4767.6 | -815.2 |
| Andorra | 115.7 | 75.3 | -40.4 | 2028.2 | 1307.7 | -720.4 |
| India | 223.3 | 188.7 | -34.6 | 4834.4 | 4115.1 | -719.4 |
| Guam | 285.1 | 207.8 | -77.3 | 5812.1 | 5096.2 | -715.9 |
| Belize | 179.1 | 136.3 | -42.8 | 3528.0 | 2834.1 | -693.9 |
| Kyrgyzstan | 302.9 | 320.6 | 17.7 | 6481.3 | 5794.4 | -686.9 |
| Paraguay | 175.9 | 144.4 | -31.5 | 3584.4 | 2924.3 | -660.1 |
| Saudi Arabia | 307.8 | 268.2 | -39.6 | 6369.9 | 5712.3 | -657.6 |
| San Marino | 121.4 | 82.4 | -39.1 | 2064.9 | 1408.3 | -656.7 |
| South Sudan | 190.3 | 166.6 | -23.7 | 4020.4 | 3392.7 | -627.7 |
| American Samoa | 247.0 | 227.7 | -19.3 | 5939.0 | 5394.5 | -544.5 |
| Ecuador | 136.2 | 118.7 | -17.5 | 2705.3 | 2187.9 | -517.3 |
| Nigeria | 199.8 | 176.8 | -23.0 | 3989.2 | 3484.3 | -504.9 |
| Jamaica | 184.6 | 147.3 | -37.4 | 3456.0 | 2955.3 | -500.7 |
| Central African Republic | 326.2 | 315.6 | -10.6 | 7380.1 | 6897.0 | -483.2 |
| Côte d'Ivoire | 223.5 | 205.9 | -17.6 | 4707.4 | 4231.0 | -476.5 |
| Mexico | 152.0 | 125.1 | -26.9 | 2820.6 | 2351.6 | -469.0 |
| Samoa | 329.0 | 310.8 | -18.1 | 7646.3 | 7182.9 | -463.4 |
| Malawi | 210.6 | 197.7 | -12.9 | 4576.1 | 4129.6 | -446.5 |
| Northern Mariana Islands | 229.4 | 216.2 | -13.3 | 5425.3 | 4991.0 | -434.3 |
| Liberia | 211.4 | 193.8 | -17.6 | 4376.4 | 3946.2 | -430.2 |
| South Africa | 171.0 | 175.2 | 4.3 | 3813.8 | 3386.7 | -427.2 |
| United States Virgin Islands | 250.8 | 231.0 | -19.8 | 4806.2 | 4379.2 | -427.0 |
| Senegal | 209.3 | 197.3 | -12.0 | 4328.3 | 3906.1 | -422.2 |
| Tokelau | 282.9 | 271.2 | -11.7 | 6578.4 | 6183.8 | -394.6 |
| Bhutan | 178.5 | 178.8 | 0.4 | 3936.6 | 3552.5 | -384.1 |
| Bangladesh | 200.5 | 193.4 | -7.1 | 4369.2 | 3999.7 | -369.6 |
| Democratic People's Republic of Korea | 238.4 | 230.9 | -7.5 | 5258.4 | 4890.1 | -368.2 |
| Eritrea | 217.0 | 224.8 | 7.8 | 5018.1 | 4650.8 | -367.3 |
| Viet Nam | 239.5 | 230.1 | -9.4 | 4838.6 | 4487.2 | -351.4 |
| Somalia | 247.0 | 244.8 | -2.2 | 5584.6 | 5240.4 | -344.2 |
| Belarus | 372.1 | 360.7 | -11.5 | 7120.2 | 6785.1 | -335.2 |
| Nepal | 156.6 | 159.9 | 3.3 | 3635.8 | 3314.9 | -320.9 |
| Mali | 195.0 | 188.2 | -6.8 | 3924.2 | 3628.8 | -295.4 |
| Uganda | 187.5 | 178.7 | -8.8 | 3844.9 | 3570.1 | -274.8 |
| Libya | 247.0 | 234.6 | -12.4 | 5155.6 | 4923.0 | -232.7 |
| Montenegro | 308.5 | 322.7 | 14.2 | 5810.3 | 5585.9 | -224.4 |
| Niger | 196.3 | 193.1 | -3.2 | 4133.1 | 3912.3 | -220.7 |
| Egypt | 431.8 | 423.5 | -8.2 | 8900.6 | 8680.5 | -220.1 |
| Tuvalu | 359.7 | 360.9 | 1.2 | 8742.0 | 8556.0 | -186.0 |
| Palau | 295.5 | 288.1 | -7.4 | 7356.2 | 7172.3 | -183.9 |
| Botswana | 257.6 | 257.5 | -0.2 | 5364.3 | 5202.8 | -161.6 |
| Sierra Leone | 232.0 | 220.9 | -11.1 | 4773.6 | 4613.7 | -159.9 |
| Benin | 207.1 | 203.3 | -3.8 | 4300.1 | 4152.6 | -147.5 |
| Togo | 219.9 | 217.3 | -2.7 | 4554.9 | 4470.2 | -84.7 |
| Djibouti | 201.7 | 206.9 | 5.2 | 4242.3 | 4181.1 | -61.2 |
| Micronesia (Federated States of) | 396.6 | 412.7 | 16.1 | 9976.3 | 9999.7 | 23.4 |
| Tonga | 174.6 | 179.6 | 4.9 | 4107.8 | 4142.3 | 34.6 |
| Guinea-Bissau | 263.3 | 277.0 | 13.8 | 5978.9 | 6018.1 | 39.2 |
| Nicaragua | 156.3 | 188.5 | 32.2 | 3075.5 | 3146.6 | 71.1 |
| Turkmenistan | 434.5 | 440.9 | 6.5 | 9003.4 | 9092.3 | 89.0 |
| Ghana | 224.3 | 238.9 | 14.6 | 4650.7 | 4753.9 | 103.2 |
| United Republic of Tanzania | 190.7 | 203.3 | 12.6 | 3946.4 | 4061.9 | 115.5 |
| Madagascar | 265.7 | 288.5 | 22.7 | 6052.0 | 6226.7 | 174.8 |
| Marshall Islands | 399.2 | 402.5 | 3.4 | 9536.8 | 9756.9 | 220.0 |
| Eswatini | 247.7 | 263.6 | 15.9 | 5105.0 | 5337.9 | 233.0 |
| Nauru | 501.7 | 519.1 | 17.3 | 12553.0 | 12817.3 | 264.3 |
| Zambia | 205.9 | 227.9 | 22.0 | 4335.7 | 4640.0 | 304.4 |
| Kenya | 151.4 | 171.7 | 20.3 | 3081.7 | 3463.5 | 381.8 |
| Chad | 179.5 | 199.6 | 20.1 | 3753.5 | 4161.9 | 408.4 |
| Cabo Verde | 161.2 | 200.0 | 38.8 | 3372.8 | 3791.3 | 418.6 |
| Indonesia | 260.8 | 305.5 | 44.7 | 5829.3 | 6283.7 | 454.4 |
| Honduras | 173.0 | 229.0 | 56.1 | 3607.9 | 4241.2 | 633.2 |
| Burkina Faso | 159.0 | 198.8 | 39.8 | 3349.6 | 4042.7 | 693.2 |
| Guinea | 174.8 | 208.0 | 33.3 | 3677.6 | 4392.5 | 715.0 |
| Cameroon | 164.9 | 198.4 | 33.4 | 3379.4 | 4107.8 | 728.4 |
| Sao Tome and Principe | 195.1 | 240.1 | 45.0 | 3998.7 | 4781.6 | 783.0 |
| Gambia | 199.4 | 242.2 | 42.8 | 4051.8 | 4892.7 | 840.9 |
| Mozambique | 220.9 | 265.9 | 45.0 | 4740.2 | 5721.2 | 981.0 |
| Zimbabwe | 204.4 | 245.8 | 41.3 | 3923.4 | 4908.5 | 985.1 |
| Azerbaijan | 373.0 | 531.5 | 158.5 | 7883.6 | 8903.6 | 1020.0 |
| Papua New Guinea | 205.3 | 248.7 | 43.4 | 4972.8 | 6076.4 | 1103.6 |
| Pakistan | 222.2 | 269.7 | 47.5 | 4937.3 | 6072.1 | 1134.9 |
| Ukraine | 414.5 | 448.5 | 33.9 | 7403.7 | 8547.1 | 1143.4 |
| Dominican Republic | 179.9 | 234.6 | 54.7 | 3667.9 | 4891.1 | 1223.2 |
| Vanuatu | 411.3 | 461.2 | 49.9 | 9845.0 | 11104.2 | 1259.2 |
| Timor-Leste | 198.3 | 277.3 | 79.0 | 4261.6 | 5623.9 | 1362.3 |
| Solomon Islands | 493.4 | 576.0 | 82.6 | 13153.7 | 14695.9 | 1542.2 |
| Lesotho | 200.8 | 286.9 | 86.1 | 4039.4 | 5919.9 | 1880.6 |
| Philippines | 160.1 | 229.3 | 69.2 | 2975.1 | 5037.9 | 2062.8 |
| Tajikistan | 305.6 | 537.3 | 231.7 | 6551.6 | 9436.8 | 2885.2 |
| Uzbekistan | 340.4 | 741.9 | 401.4 | 6812.4 | 12713.9 | 5901.5 |
| ASR(per 100,000 population) , age standardized rate; DALYs, Disability-Adjusted Life Years | | | | | | |

Table S4 Metabolism-related CVDs deaths/DALYs ASR across 21 GBD world regions and SDI in 1990 and 2019.

| location | 1990 | | | | 2019 | | |
| --- | --- | --- | --- | --- | --- | --- | --- |
|  | SDI | deaths ASR | | DALYs ASR | SDI | deaths ASR | DALYs ASR |
| Global | 0.5 | 252.9 | 4965.8 | | 0.7 | 176.1 | 3573.5 |
| Oceania | 0.4 | 265.1 | 6417.0 | | 0.5 | 283.4 | 6882.1 |
| South Asia | 0.3 | 219.3 | 4777.3 | | 0.5 | 194.9 | 4254.3 |
| Central Latin America | 0.5 | 175.0 | 3388.2 | | 0.6 | 126.9 | 2439.5 |
| Caribbean | 0.5 | 238.1 | 4867.2 | | 0.6 | 180.4 | 3822.8 |
| Andean Latin America | 0.5 | 127.3 | 2577.6 | | 0.6 | 88.4 | 1712.7 |
| Tropical Latin America | 0.5 | 255.6 | 5404.8 | | 0.6 | 129.4 | 2764.9 |
| Southeast Asia | 0.5 | 234.8 | 5077.0 | | 0.6 | 223.0 | 4730.4 |
| North Africa and Middle East | 0.4 | 373.3 | 7709.0 | | 0.7 | 289.7 | 5711.2 |
| Central Asia | 0.6 | 384.8 | 7816.2 | | 0.7 | 454.0 | 8507.1 |
| East Asia | 0.5 | 237.9 | 4591.9 | | 0.7 | 192.2 | 3512.3 |
| Southern Latin America | 0.6 | 204.4 | 3920.5 | | 0.7 | 117.5 | 2216.5 |
| Central Europe | 0.6 | 412.0 | 7769.4 | | 0.8 | 238.3 | 4181.2 |
| Eastern Europe | 0.7 | 421.9 | 7898.4 | | 0.8 | 352.4 | 6752.7 |
| Australasia | 0.7 | 209.4 | 3776.8 | | 0.8 | 76.9 | 1331.9 |
| Western Europe | 0.8 | 216.3 | 3828.5 | | 0.8 | 90.7 | 1535.2 |
| High-income North America | 0.8 | 206.1 | 3992.6 | | 0.9 | 108.4 | 2199.0 |
| High-income Asia Pacific | 0.8 | 154.1 | 2822.3 | | 0.9 | 50.9 | 1046.0 |
| SDI, social-demographic index; ASR(per 100,000 population) , age standardized rate; DALYs, Disability-Adjusted Life Years | | | | | | | |

Table S5 Number and ASR of metabolism-related CVDs deaths/DALYs/YLDs/YLLs from 1990 to 2029.

| Year | Number(millions) | | | | ASR | | | |
| --- | --- | --- | --- | --- | --- | --- | --- | --- |
|  | deaths | DALYs | YLDs | YLLs | deaths | DALYs | YLDs | YLLs |
| 1990 | 8.6 | 192.7 | 11.0 | 181.7 | 252.9 | 4965.8 | 278.9 | 4686.9 |
| 1991 | 8.7 | 195.3 | 11.2 | 184.1 | 249.9 | 4914.7 | 277.6 | 4637.1 |
| 1992 | 8.9 | 199.1 | 11.4 | 187.7 | 248.0 | 4893.5 | 276.4 | 4617.1 |
| 1993 | 9.2 | 205.3 | 11.6 | 193.7 | 249.5 | 4931.2 | 275.3 | 4655.9 |
| 1994 | 9.4 | 209.7 | 11.9 | 197.9 | 247.9 | 4917.3 | 274.4 | 4642.8 |
| 1995 | 9.5 | 211.2 | 12.1 | 199.1 | 243.9 | 4840.7 | 273.9 | 4566.8 |
| 1996 | 9.5 | 212.3 | 12.4 | 200.0 | 239.4 | 4753.7 | 273.5 | 4480.1 |
| 1997 | 9.6 | 214.3 | 12.7 | 201.6 | 235.5 | 4683.1 | 273.3 | 4409.8 |
| 1998 | 9.7 | 215.9 | 13.0 | 202.9 | 231.7 | 4606.4 | 273.0 | 4333.4 |
| 1999 | 9.9 | 220.1 | 13.3 | 206.8 | 230.2 | 4581.3 | 272.8 | 4308.6 |
| 2000 | 10.1 | 224.0 | 13.6 | 210.4 | 227.8 | 4547.2 | 272.5 | 4274.7 |
| 2001 | 10.2 | 227.5 | 13.9 | 213.6 | 225.4 | 4505.2 | 272.1 | 4233.1 |
| 2002 | 10.5 | 232.4 | 14.2 | 218.2 | 224.4 | 4489.4 | 271.3 | 4218.1 |
| 2003 | 10.6 | 235.6 | 14.5 | 221.2 | 221.7 | 4440.0 | 270.3 | 4169.7 |
| 2004 | 10.7 | 236.2 | 14.8 | 221.4 | 216.0 | 4336.1 | 269.4 | 4066.7 |
| 2005 | 10.9 | 239.9 | 15.1 | 224.8 | 213.5 | 4292.0 | 268.9 | 4023.1 |
| 2006 | 10.9 | 240.0 | 15.5 | 224.5 | 207.6 | 4180.7 | 268.7 | 3912.0 |
| 2007 | 11.0 | 242.3 | 16.0 | 226.4 | 203.7 | 4107.7 | 268.7 | 3839.0 |
| 2008 | 11.2 | 246.8 | 16.4 | 230.5 | 201.8 | 4073.1 | 268.7 | 3804.4 |
| 2009 | 11.4 | 249.0 | 16.8 | 232.1 | 198.2 | 3999.7 | 268.9 | 3730.8 |
| 2010 | 11.6 | 253.4 | 17.3 | 236.1 | 196.2 | 3963.5 | 269.1 | 3694.4 |
| 2011 | 11.8 | 256.5 | 17.8 | 238.7 | 193.0 | 3904.0 | 269.4 | 3634.6 |
| 2012 | 11.9 | 259.9 | 18.3 | 241.6 | 189.9 | 3847.0 | 269.8 | 3577.2 |
| 2013 | 12.1 | 262.7 | 18.8 | 243.9 | 187.2 | 3785.9 | 270.4 | 3515.5 |
| 2014 | 12.3 | 265.5 | 19.4 | 246.1 | 184.3 | 3724.0 | 271.0 | 3453.0 |
| 2015 | 12.6 | 271.7 | 20.0 | 251.7 | 183.0 | 3708.9 | 271.6 | 3437.3 |
| 2016 | 12.9 | 276.3 | 20.6 | 255.7 | 180.8 | 3671.8 | 272.8 | 3399.1 |
| 2017 | 13.1 | 280.0 | 21.3 | 258.8 | 178.2 | 3623.5 | 273.9 | 3349.6 |
| 2018 | 13.4 | 285.3 | 21.9 | 263.5 | 177.1 | 3596.5 | 274.4 | 3322.1 |
| 2019 | 13.7 | 291.0 | 22.5 | 268.5 | 176.1 | 3573.5 | 274.9 | 3298.6 |
| 2020 | 14.0 | 294.3 | 23.1 | 271.5 | 173.4 | 3531.7 | 275.4 | 3256.8 |
| 2021 | 14.3 | 297.7 | 23.7 | 274.5 | 170.8 | 3483.9 | 275.8 | 3209.1 |
| 2022 | 14.6 | 301.1 | 24.3 | 277.5 | 168.1 | 3436.0 | 276.3 | 3161.4 |
| 2023 | 14.8 | 304.5 | 25.0 | 280.5 | 165.5 | 3388.2 | 276.8 | 3113.7 |
| 2024 | 15.1 | 307.9 | 25.6 | 283.4 | 162.8 | 3340.3 | 277.3 | 3066.0 |
| 2025 | 15.4 | 311.3 | 26.2 | 286.4 | 160.2 | 3292.5 | 277.7 | 3018.3 |
| 2026 | 15.7 | 314.7 | 26.8 | 289.4 | 157.5 | 3244.6 | 278.2 | 2970.6 |
| 2027 | 15.9 | 318.1 | 27.4 | 292.4 | 154.9 | 3196.8 | 278.7 | 2922.9 |
| 2028 | 16.2 | 321.5 | 28.0 | 295.4 | 152.2 | 3148.9 | 279.2 | 2875.2 |
| 2029 | 16.5 | 324.8 | 28.7 | 298.4 | 149.6 | 3101.1 | 279.6 | 2827.5 |
| ASR(per 100,000 population) , age standardized rate; CVDs, cardiovascular disease; DALYs, Disability-Adjusted Life Years; YLDs, Years Lived with Disability; YLLs, Years of Life Lost. | | | | | | | | |
